# Survival of patients with metastatic renal cell carcinoma with or without brain metastases

**DOI:** 10.1093/oncolo/oyaf387

**Published:** 2025-11-25

**Authors:** Noam Savion-Gaiger, Bryden Considine, Nitzan Hasson, Melanie Nelson, Veronica Chiang, Harriet M Kluger, David A Braun, David Schoenfeld, Mario Sznol, Michael S Leapman, Michael E Hurwitz

**Affiliations:** Yale Cancer Center, New Haven, CT 06520, United States; Yale Cancer Center, New Haven, CT 06520, United States; Yale Cancer Center, New Haven, CT 06520, United States; Yale Cancer Center, New Haven, CT 06520, United States; Department of Neurosurgery, Yale School of Medicine, New Haven, CT 06510, United States; Yale Cancer Center, New Haven, CT 06520, United States; Yale Cancer Center, New Haven, CT 06520, United States; Yale Cancer Center, New Haven, CT 06520, United States; Yale Cancer Center, New Haven, CT 06520, United States; Department of Urology, Yale School of Medicine, New Haven, CT 06520, United States; Yale Cancer Center, New Haven, CT 06520, United States

**Keywords:** renal cell carcinoma, brain metastases, immune checkpoint inhibitors, real-world data

## Abstract

**Background:**

Historically, brain metastasis (BM) is associated with poor survival for patients with metastatic renal cell carcinoma (mRCC). The prognostic significance of BM in the immune checkpoint (ICI) era is unclear.

**Methods:**

We performed a retrospective cohort study of patients diagnosed with clear cell RCC (ccRCC) between 2012-2023. We examined the association between BM and overall survival (OS) among patients by treatment type (with or without ICI in any line of therapy) and whether they had brain MRI screening.

**Results:**

We identified 338 patients with metastatic ccRCC between 2012 and 2023, of whom 96 (28.4%) had BM. mOS from the time of metastatic ccRCC diagnosis was 54.3 months (mo) in patients without BM versus 37.3 mo in patients with BM (*P* = .03). Among patients who received ICI therapy, mOS was 66.4 mo in those without BM versus 37.7 mo in those with BM (*P* = .01). In patients who did not receive ICI therapy, mOS was 32.1 mo in those without BM versus 17.6 mo in those with BM (*P* = .4). In those with BM, mOS from the time of initial metastatic disease diagnosis was 86.7 mo for those who underwent MRI brain screening versus 27.9 mo for those who did not (*P* = .00016).

**Conclusion:**

In our ccRCC population, OS for patients with metastatic ccRCC and brain metastases has improved in the era of ICI therapy. Brain metastases are associated with poor prognosis. Patients with brain metastases discovered on screening had improved overall survival compared to those with brain metastases discovered because of symptoms.

Implications for practice In this ccRCC population, there is a higher prevalence of brain metastases than previously thought (>28%). Overall survival (OS) for patients with metastatic ccRCC and brain metastases has improved in the era of immune checkpoint inhibitor (ICI) therapy. Patients with brain metastases discovered on screening had improved OS compared to those discovered from symptoms. Screening Brain MRIs should be a standard of care in mRCC.

## Introduction

Brain metastasis (BM) is associated with a poor prognosis in renal cell carcinoma (RCC).^1–6^ The true prevalence of BM in metastatic RCC (mRCC) is unknown but incidence has been estimated between 8% to 28% in recent studies.^2^^,^^3^^,^^7–9^

Overall survival has improved for patients with mRCC and BM due to advances in both systemic and localized therapies with median overall survival (mOS) reported to range from 8.4-14.4 mo.^2^^,^^3^^,^^5^^,^^6^ The current management of BM is dependent on the number, size, and location of the metastases and is often a combination of both local and systemic therapies. Craniotomy, stereotactic radiosurgery (SRS), and whole brain radiation (WBRT) are the most effective local interventions in the management of BM.^10^ Systemic therapies have shown limited benefit. Sunitinib has not demonstrated significant efficacy against BM though two studies reported intracranial activity and improved outcomes with cabozantinib in patients with BM.^11^^,^^12^ The role of ICIs in this setting remains uncertain, as patients with BM are frequently excluded from pivotal clinical trials, and prospective data are lacking.^13^ However, a retrospective subgroup analysis of RCC patients with BM found that ICI treatment was associated with improved survival (77.2 mo with ICIs versus 25.2 mo without; *P* < .001).^14^ Additionally, one study demonstrated improved OS with ICI/TKI combination therapy compared with either treatment alone in patients with RCC and BM. Conversely, preliminary post hoc analyses from JAVELIN Renal 101 reported comparable poor progression-free survival (PFS) between axitinib plus avelumab and sunitinib alone in patients with BM. The intracranial activity of the immune checkpoint inhibitor (ICI) nivolumab was evaluated in 34 patients with only four (12%) with an intracranial response.^15^ However, this study included a heavily pre-treated population, and the extracranial response rate was only 21%, and studies in other tumor types with anti-PD-1 monotherapy suggest that intra- and extra-cranial responses are largely concordant.^16–18^ Early results from CheckMate 920 (NCT02982954) were more encouraging, with ipilimumab plus nivolumab in 28 patients with mRCC and asymptomatic BM yielding a median PFS of 9 months and a systemic ORR of 28.6%.^19^

In this study, we investigate patient characteristics, management, and outcomes of patients with clear cell mRCC and BM in the modern era of ICI therapy. We compared outcomes of patients with and without BM at a single National Cancer Institute-designated comprehensive cancer center between 2012-2023. Specifically, we sought to assess outcomes of patients who received ICI as a component of their therapy with those who did not. By further understanding this population, we intended to identify areas in which the management of patients with BM can be improved.

## Methods

We performed a retrospective study of patients diagnosed with metastatic ccRCC at the Yale Cancer Center between 01/2012-12/2023. This timeframe reflects the contemporary treatment era and encompasses patients who were treated with both tyrosine kinase inhibitors (TKIs) and ICIs. We collected demographic data related to sex, race/ethnicity, age at initial RCC diagnosis, age at mRCC diagnosis, and age at mRCC BM diagnosis. Clinicopathologic data were collected through a review of medical charts and included histology, tumor grade, International Metastatic Renal Cell Carcinoma Database Consortium (IMDC) score, size and number of BM, whether patient developed symptoms at the onset of BM, whether screening brain MRI imaging was performed, treatments including local (nephrectomy), systemic and brain targeted therapies. We defined patients as undergoing screening if a brain MRI was performed within 6 mo preceding the diagnosis of mRCC BM in the absence of symptoms. A cut-off date of May 31, 2024, was used for both the development of BM and for overall survival (OS). Patients with a concurrent metastatic malignancy and those who were lost to follow-up prior to their deaths or the cut-off date were excluded. This study was conducted with the approval of the Institutional Review Board of Yale ­University, protocol numbers HIC#0608001773 and HIC#0805003787.

We identified 529 patients with a diagnosis of mRCC made between 1/2012-12/2023, 404 (76.4%) of whom had clear cell histology. We excluded those without complete treatment histories or concurrent malignancies, resulting in 338 evaluable patients (Supplemental Figure S1). We further characterized which local and systemic therapies a patient received and whether the systemic therapy included an ICI (anti PD-1/PD-L1 or CTLA-4) or not. Patients receiving non-ICI therapy included all other systemic therapies exclusively including TKI therapy and IL-2. The timing and duration of systemic therapies was recorded in relationship to the development of BM.

### Statistical analysis

We compared clinical and demographic characteristics among patients based on brain metastasis status using chi-square tests and Student’s *T*-tests or Wilcoxon test. We used the Kaplan–Meier method to compare OS between patients with BM. Analyses were conducted on the entire cohort, and within subsets of patients by treatment (ie, ICI containing regimen versus non-ICI therapy). The log-rank test was used for evaluation of OS.

We performed multivariate logistic regression analysis to evaluate associations between demographic and clinical features and the risk to develop BM, reported by odds ratio and 95% confidence intervals (95% CI). To evaluate the independent effects of clinical and pathological factors on overall survival, we performed a multivariable Cox Proportional Hazards regression analysis. We reported hazard ratios (HRs) with 95% confidence intervals (CIs) to quantify the association between each variable and survival outcomes. Descriptive data, comparative analyses, and overall survival (OS) were performed using R (version 4.4.1).

## Results

We identified 338 evaluable patients with clear cell mRCC between 2012-2023. 96 (28.4%) were diagnosed with BM at any point during their illness. The mean age at diagnosis of mRCC was 64.3 years, and at development of BM was 62.5 years. Most patients were male (75.1% [254/338] of all patients; 76.0% (73/96) of BM patients), and non-Hispanic (90.5% [306/338] of all patients; 95.8% [92/96] of brain met patients) (Table 1). IMDC Intermediate-risk was the most frequent in both groups with 55.8% in non-BM and 55.2% in BM. The median OS from time of metastasis development for the entire mRCC cohort was 47.4 mo (95% CI, 38.3-60.7) (Supplemental Figure S2). There was no statistical difference in the distribution of IMDC scores, grade of tumor at time of nephrectomy, number receiving nephrectomy or systemic therapy received between patients with or without BM (Table 1). Median OS from time of diagnosis of mRCC based on IMDC score groups differed: 23.2 mo (95%CI 16.5-33.9), 54.0 mo (95% CI 38.2-76.7) 149.8 mo (95%CI 83.0-NR) for poor, intermediate and favorable risk, respectively (*P* < .001) (Supplemental Figure S3).

**Table 1.** Demographic and clinical characteristics of the cohort (*n* = 338).

		Brain metastases	Non-brain metastases	Total	*P*-value
		*n* = 96	*n* = 242	*n* = 338	
**Age at metastases diagnosis (years)**					**<.0001**
	Mean ± SD	61.1 ± 10.5	65.6 ± 11.3	64.3 ± 11.3	
**Gender**					.9
	Male	73 (76.0%)	181 (74.8%)	254 (75.1%)	
	Female	23 (23.9%)	61 (25.2%)	84 (24.9%)	
**Ethnicity**					.06
	Non-Hispanic	92 (95.8%)	214 (88.4%)	306 (90.5%)	
	Hispanic or Latino	1 (1.0%)	18 (7.4%)	19 (5.6%)	
	Unknown	3 (3.1%)	10 (4.1%)	13 (3.8%)	
**Race**					.4
	White or Caucasian	84 (87.5%)	195 (80.5%)	279 (82.5%)	
	African American	6 (6.2%)	13 (5.3%)	19 (5.6%)	
	Asian	1 (1.0%)	6 (2.4%)	7 (2.1%)	
	Unknown	5 (5.2%)	28 (11.8%)	32 (9.4%)	
**Nephrectomy**					.1
	Yes	70 (72.9%)	194 (80.1%)	264 (78.1%)	
	No	26 (27.1%)	48 (19.9%)	74 (21.9%)	
**Grade**					.1
	1	3 (3.1%)	4 (1.6%)	7 (2.1%)	
	2	10 (10.4%)	48 (19.8%)	58 (17.2%)	
	3	35 (36.5%)	71 (29.3%)	106 (31.4%)	
	4	25 (26.0%)	56 (23.1%)	81 (24.0%)	
**IMDC score** ^a^					.2
	Poor	21 (21.8%)	49 (20.2%)	70 (20.7%)	
	Intermediate	53 (55.2%)	135 (55.8%)	188 (55.6%)	
	Favorable	7 (7.3%)	34 (14.0%)	41 (12.1%)	
	Unknown	15 (15.6%)	24 (9.9%)	39 (11.5%)	
**1st line systemic therapy**					.1
	ICIs^b^	41 (42.7%)	113 (46.6)	154 (45.6%)	
	TKI^c^	30 (31.2%)	65 (26.8%)	95 (28.1%)	
	ICIs^b^ + TKI^c^	3 (3.1%)	10 (4.1%)	13 (3.8%)	
	Other^d^	17 (17.7%)	25 (10.3%)	42 (12.4%)	
	No systemic therapy	5 (5.2)%)	29 (11.9%)	34 (10.1%)	
**History of systemic ICI** ^b^ **treatment**					.1
	ICIs^b^	77 (80.2%)	170 (70.2%)	247 (73.0%)	
	Never treated with ICIs^b^	14 (14.5%)	43 (17.7%)	57 (16.8%)	
	No systemic treatment	5 (5.2%)	29 (11.9%)	34 (10.1%)	

a IMDC score: International Metastatic RCC Database Consortium Score.

b ICI: Immune check-point inhibitors.

c TKI: Tyrosine Kinase inhibitors.

d Other treatments includes: mTOR Inhibitors, IL-2 Therapy, VEGF inhibitor.

**Figure 1.**
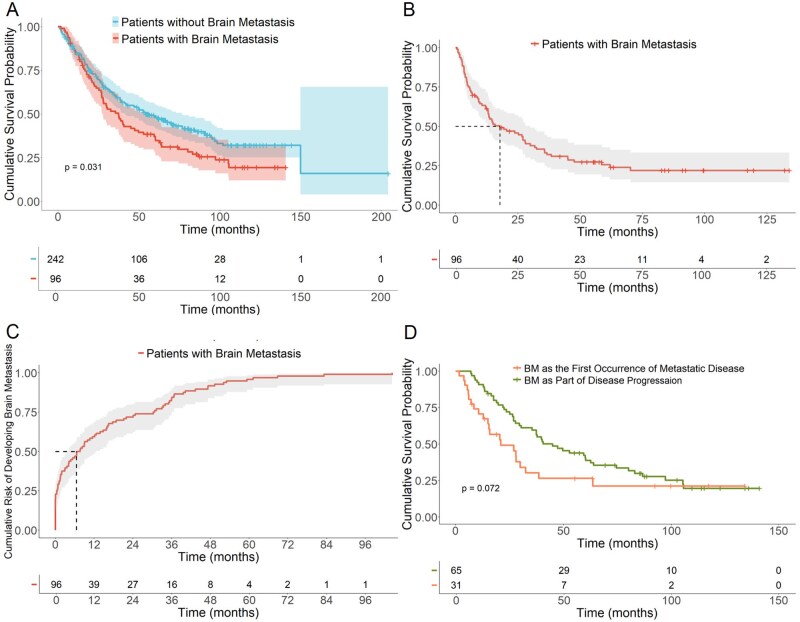
(A) Overall survival of patients with metastatic disease with or without brain metastases from time of diagnosis of metastatic disease. Shaded areas represent 95% confidence intervals. (B) Overall survival of patients with brain metastases from time of diagnosis of brain metastases. (C) Timing of development of brain metastases after diagnosis of metastatic cancer. (D) Overall survival of patients with brain metastases as first evidence of metastatic cancer versus patients who developed brain metastases after diagnosis of other metastases.

From the time of first diagnosis of mRCC, median OS was 54.3 mo (95% CI, 41.5-76.7) in patients who never developed BM versus 37.3 mo (95% CI, 27.8-52.8) in patients who developed BM at some point in the course of their illness (*P* = .03), demonstrating worse overall survival for patients who develop BM (Figure 1A). The median OS from the time of BM development was 17.8 mo (95% CI, 13.1-29.9) (Figure 1B). Among all patients with mRCC BM, 20.8% (20/96) presented with BM at the time of original kidney cancer diagnosis. An additional 11.4% (11/96) presented with BM at the first occurrence of metastatic disease. Most patients, 67.7% (65/96), developed BM as a site of progression after other systemic disease. Among those who developed BM after other systemic disease (*n* = 65), 12.3% (8/65) occurred within 3 mo and 40.0% (26/65) within 1 year of diagnosis of mRCC (Table 2). Brain was the sole site of metastasis in 8.3% cases (8/96), all presented with symptomatic BM. The median time to development of BM was 6.5 mo (95% CI, 4.1-12.6) (Figure 1C). From the time of mRCC there was no significant difference in mOS among patients who developed BM as the first site of metastatic disease versus those in whom BM developed as progression of metastatic disease (20.9 mo (95%CI: 15.0-38.5) versus 44.9 mo (95%CI: 35.4-63.9); *P* = .07) though there appears to be a trend towards worse survival (Figure 1D).

**Table 2.** Clinical and radiological characteristics of patients with brain metastasis (*n* = 96).

**Age at RCC** ^a^ **Brain metastasis diagnosis (years)**		
	Mean ± SD	62.5 ± 10.7
**Brain metastasis at RCC** ^a^ **diagnosis**		
	Yes	20 (20.8%)
	No	76 (79.1%)
**Brain metastasis as the initial site of metastatic disease**		
	Yes	31 (32.2%)
	No	65 (67.7%)
**Brain metastasis as part of disease progression (*n* = 65)**		
	Diagnosed within 3 months of mRCC^b^ diagnosis	8 (12.3%)
	Diagnosed within 12 months of mRCC^b^ diagnosis	26 (40.0%)
**Screening brain MRI** ^c^		
	Yes	68 (70.8%)
	No	28 (29.1%)
**Number of brain metastasis**		
	Single	40 (41.6%)
	>1	56 (58.3%)
**Clinical presentation of brain metastasis**		
	Symptomatic	57 (59.3%)
	Asymptomatic	39 (40.6%)
**Brain targeted therapy**		
	GKRS^d^	76 (79.1%)
	WBRT^e^	13 (13.5%)
	Craniotomy	20 (20.8%)
	None	10 (10.4%)

a RCC: Renal Cell Carcinoma.

b mRCC: Metastatic Renal Cell Carcinoma.

c Screening brain MRI: brain MRI performed within 6 months prior to brain metastasis diagnosis.

d GKRS: Gamma Knife Radiosurgery,

e WBRT: Whole brain radiotherapy.

**Figure 2.**
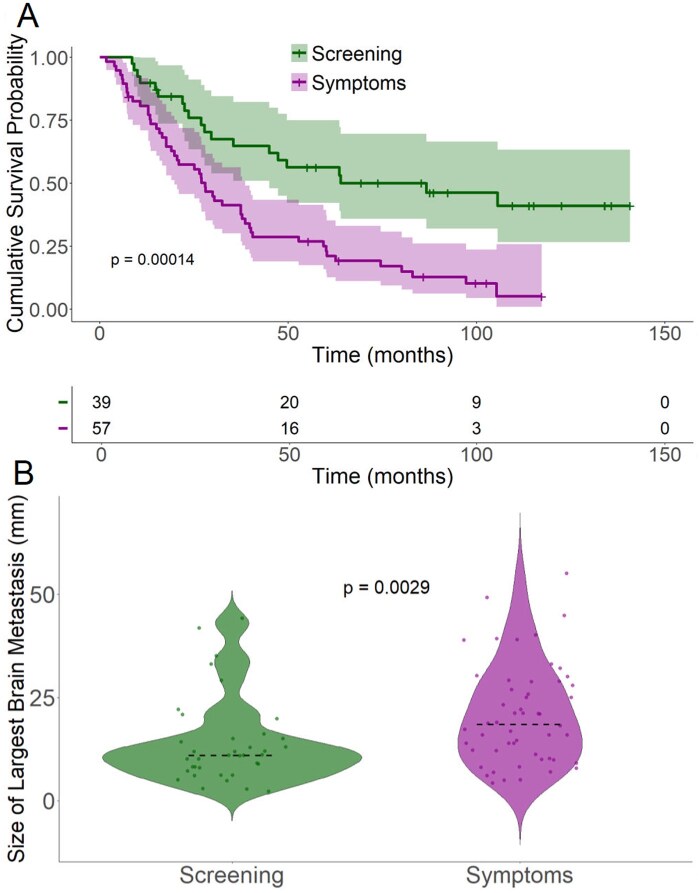
(A) Overall survival of patients with brain metastases from time of diagnosis of metastatic disease in patients who had screening brain MRI versus those who did not. (B) Size of largest brain metastasis at diagnosis in patients who were diagnosed by screening brain MRI versus those who were diagnosed by the development of symptoms.

Of patients with RCC BM, 59.3% (57/96) of patients had CNS symptoms, which led to brain imaging making the diagnosis of BM (Table 2). From the time of mRCC, patients who had CNS symptoms at the diagnosis of BM had a worse overall survival than those with asymptomatic BM who were diagnosed with a screening MRI (27.9 mo (95% CI 19.8-38.5) vs 86.7 mo (95%CI 44.9-NR), *P* = .00016) (Figure 2A). In patients in whom the diagnosis was made based on the presence of symptomatic BM, the median size of the largest brain metastasis was 18.5 mm (4.0-55.0) versus 11.0 mm (2.0-44.0) in those who were asymptomatic (*P* = .0029) (Figure 2B). There was no statistical difference in the time of development of BM from diagnosis of metastatic disease or in the number of patients with >1 BM in either group (*P* = .9 and *P* = .7, respectively) (Supplemental Figure S4 and Supplementary Table S1, respectively). 40/96 patients (41.7%) had a single BM at diagnosis, with an average size of 19.2 ± 12.2 mm. Among these, 13 (32.5%) underwent craniotomy, 32 (80.0%) received Gamma Knife radiosurgery (GKRS), and 3 (7.5%) were treated with whole-brain radiotherapy (WBRT) (Table 2). There was no statistically significant difference in OS between patients with a single BM and those with multiple BMs (Figure S5). To assess whether any features were associated with the development of BM, univariate and multivariate logistic regression were performed (age at metastatic disease, gender, ethnicity, race, nephrectomy status, grade of primary, ICI treatment, IMDC score). None of the variables demonstrated a significant association with an increased risk of developing BM. Age at mRCC diagnosis had a modest yet statistically significant protective effect with an odds ratio of 0.95 (95% CI: 0.92-0.99, *P* = .005), indicating a minor reduction in BM risk with advancing age, even after adjusting for other covariates. While Hispanic ethnicity showed a weakly significant association with reduced BM risk in a univariate analysis (OR: 0.1, 95% CI: 0.01-0.99), this effect lost significance after adjusting for other variables (Supplementary Table S3).

We conducted univariate and multivariate Cox proportional hazards analyses to evaluate predictive and prognostic factors influencing overall survival (OS) in patients. The univariate analysis indicated that BM, poor IMDC score and lack of ICI treatment were associated with a higher risk of mortality while nephrectomy, favorable and intermediate IMDC scores were linked to improved survival (Table 3). In the multivariate analysis, BM, poor IMDC score and non-ICI treatment remained significantly associated with worse survival outcomes, with HRs of 1.5 (95% CI: 1.1-2.2, *P* = .02), 2.7(95%CI: 1.4-5.0, *P* = .002) and 1.7 (95% CI: 1.1-2.7, *P* = .02), respectively. Nephrectomy remained associated with better survival outcomes with HR of 0.3 (0.1-0.6, *P* = .001). Further analysis of individual IMDC components as independent predictors revealed that only Karnofsky Performance Status (KPS) < 80 maintained a significant association with OS in the multivariate model (HR 3.6; 95% CI: 2.3-5.6; *P* < .001). Other IMDC factors (treatment initiation within one year, hemoglobin (Hb) < 12 g/dL, polymorphonuclear leukocytes (PMN) > 7,000/mL, calcium (Ca) > 10.2 mg/dL, and platelet count (PLT) < 150 × 10^9^/L) did not show significant associations with OS in either univariate or multivariate analyses.

**Table 3.** Comparative univariate and multivariate Cox proportional hazards analysis of prognostic factors influencing overall survival.

		Univariate		Multivariate	
		Hazard ratio (95% CI)	*P*-value	Hazard ratio (95% CI)	*P*-value
**Age at metastasis diagnosis**		1.0 (0.99-1.02)	.1	1.0 (1.0-1.04)	**.03**
**Brain metastases**					
	No (*n* = 242)	Reference			
	Yes (*n* = 96)	1.3 (1.0-1.8)	**.03**	1.5 (1.1-2.2)	**.02**
**Gender**					
	Male (*n* = 254)	Reference			
	Female (*n* = 84)	1.0 (0.7-1.4)	.7	1.0 (0.7-1.6)	.8
**Ethnicity**					
	Hispanic (*n* = 19)	Reference			
	Non-Hispanic (*n* = 306)	0.8 (0.5-1.5)	.5	0.5 (0.1-1.6)	.2
	Unknown (*n* = 13)	0.99 (0.4-2.5)	.9	0.6 (0.1-2.5)	.5
**Race**					
	Asian (*n* = 7)	Reference			
	White or Caucasian (*n* = 279)	0.9 (0.3-2.5)	.9	0.7 (0.2-2.4)	.6
	Black or African American (*n* = 19)	2.1 (0.7-6.3)	.2	1.8 (0.5-6.8)	.4
	Unknown (33)	0.8 (0.3-2.3)	.6	0.5 (0.1-2.3)	.4
**Nephrectomy**					
	No (*n* = 74)	Reference			
	Yes (*n* = 264)	0.3 (0.2-0.5)	**.0001**	0.3 (0.1-0.6)	**.001**
**Grade**					
	Grade 1 (*n* = 7)	Reference			
	Grade 2 (*n* = 58)	1.6 (0.5-5.2)	.4	1.5 (0.5-5.1)	.5
	Grade 3 (*n* = 106)	1.8 (0.6-5.9)	.3	1.9 (0.6-6.0)	.3
	Grade 4 (*n* = 81)	1.9 (0.6-6.0)	.3	1.5 (0.5-5.0)	.5
**Systemic treatment**					
	ICIs^a^ only (*n* = 247)	Reference			
	Non-ICIs^a^ only (*n* = 57)	1.9 (1.4-2.7)	**.0001**	1.7 (1.1-2.7)	**.02**
	No systemic treatment (*n* = 34)	0.7 (0.4-1.2)	.2	1.2 (0.6-2.5)	.6
**IMDC Score** ^b^					
	Unknown (*n* = 39)	Reference			
	Favorable (*n* = 41)	0.4 (0.2-0.7)	**.002**	0.6 (0.3-1.3)	.2
	Intermediate (*n* = 188)	0.9 (0.6-1.3)	.6	1.3 (0.8-2.3)	.3
	Poor (*n* = 70)	1.7 (1.0-2.7)	**.03**	2.7 (1.4-5.0)	**.002**

a ICI: Immune check-point inhibitors.

b IMDC score: International Metastatic RCC Database Consortium Score.

**Figure 3.**
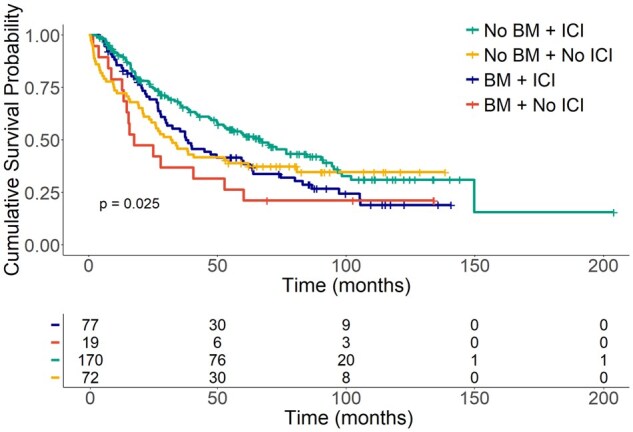
Overall survival of patients with or without brain metastases stratified by receipt of ICI treatments.

To investigate the association of different systemic therapies with outcomes in patients with and without BM we classified patients based on whether they received ICI or not since the diagnosis of mRCC. The demographics of patients who received ICI versus those who did not, and of patients who were screened versus those who were not, are presented in Tables S1 and S2,respectively. Among all patients with mRCC, 73.0% (247/338) received ICI therapy, 16.8% (57/338) only non-ICI therapy and 10.0% (34/338) received no systemic therapy. In patients with BM 80.2% (77/96) received ICI therapy, 14.5% (17/96) non-ICI therapy and 5.2% (5/96) received no systemic therapy (Table 1). Survival analysis from the time of mRCC was performed and stratified based on the presence of BM and ICI therapy (Figure 3). Treatment with ICIs approximately doubled median OS in patients with or without BM; however, the difference was statistically significant only in patients without BM (*P* = .002) (Table 4). The presence of BM was associated with shorter OS in both ICI-treated and non-ICI-treated patients, but statistical significance was reached only among those who received ICI (*P* = .01) (Table 4). In patients with BM, we further stratified systemic therapies based on when it was given in relation to the development of BM. Following the diagnosis of BM, 57.7% of patients received ICI, 23.9% non-ICI and 18.3% no systemic therapy. Local therapies for BM were frequently used with 78.9% receiving SRS.10/96 (10.4%) patients did not undergo local therapy for BM (Table 2). Among them, 7 received ICI treated and 5 died within 3 mo of diagnosis of BM. Patients treated with SRS had better OS than those receiving WBRT; however, this difference was not statistically significant, likely due to the small sample size (Figures S6 and S7).

**Table 4.** mOS from time of metastatic ccRCC^a^ diagnosis, stratified by presence of brain metastases and Immune Checkpoint Inhibitor treatment.

	Brain metastases *n* = 96	No brain metastases *n* = 242	*P*-value
	Median OS (95% CI)	Median OS (95% CI)	
**Received ICI** ^b^ **treatment (*n* = 247)**	*n* = 77 37.7 (29.6-63.6)	*n* = 170 66.4 (50.1-91.0)	**.01**
**Did not receive ICI** ^b^ **treatment (*n* = 91)**	*n* = 19 17.6 (14.6-NA^c^)	*n* = 72 32.2 (23.4-80.7)	.4
***P*-value**	.1	**.002**	

a ccRCC: Clear Cell Renal Cell Carcinoma.

b ICI: Immune check-point inhibitors.

c The standard error median survival time are not reported due to a high level of censoring, which limits the estimation to the largest observed survival time.

## Discussion

We evaluated patients diagnosed with mRCC over a 12-year period between 2012-2023 at a NCI-designated cancer center. We identified 96 patients with BM out of the 338 patients with ccRCC, the majority of whom were diagnosed in the ICI era. To our knowledge this is the largest cohort of patients in this timeframe that compares outcomes in patients with and without BM. We found that overall survival improved for patients with mRCC including in the subset with BM. Nonetheless, BM remained an important adverse prognostic factor associated with significantly poorer survival.

Outcomes for patients with metastatic ccRCC have improved in the past two decades, first with the use of TKIs and subsequently the use of ICI and combination ICI/TKI therapies.^20–23^ Patients with active or symptomatic BM have been excluded from these large clinical trials and the effect of ICI on patients with BM is not well known. Historically, patients with BM have done poorly with reported median overall survival of 8.4-14.4 mo from diagnosis of BM.^2^^,^^3^^,^^5^^,^^6^ In our study, we observed a mOS of 17.8 mo from the diagnosis of BM and 37.3 mo from the time of diagnosis of metastatic disease. To our knowledge this is the longest reported mOS in patients with BM. This improvement in mOS might reflect advances in systemic therapy, local therapy, increased screening or a combination of all these factors. When comparing survival between patients who presented with BM as first site of metastasis to those who presented with BM as disease progression, there was a trend towards worse survival in patients whose first site of metastasis was the brain, though this was not statistically significant (20.9 mo vs 44.9 mo, *P* = .07). OS was improved among patients with BM who were diagnosed through screening compared to those diagnosed after developing BM-related symptoms. Previous studies have reported that substantial proportions of patients with metastatic RCC may have occult BM and recommended considering brain screening, particularly in patients with a high metastatic burden or progression after first-line treatment.^24^

In multivariable analysis only BM, nephrectomy, treatment with ICIs and IMDC score were associated with survival; thus, baseline features were largely not prognostic in our analysis.

A striking finding from this study is the substantial difference in OS between patients who received ICI versus those who did not. Specifically, In those without BM, mOS was 66.4 mo in those who received ICI therapy vs 32.2 mo in those who did not (*P* = .002) and in those with BM, mOS was 37.7 mo in those who received ICI therapy vs 17.6 mo in those who did not (*P* = .1). While the difference for those without BM was not statistically significant, the number of patients who did not receive ICI was small, so conclusions are hard to make. These real-world data underscore improvements in OS attributable to ICI for both patients with and without BM.

The precise reasons for worse survival among patients with BM in the ICI era are not clear. Historically, this was attributed to poor CNS disease control as prior to the widespread use of SRS, the BM recurrence rates were 31-70% in patients with craniotomy and 39-52% with WBRT.^10^ Now, recurrence rates are 0-15% with SRS and as low as 4.2% in those with craniotomy followed by SRS.^10^ One of the most striking findings in this study is the large difference in mOS between asymptomatic patients screened by MRI for BMs versus those whose BMs presented with symptomatology (OS 86.7 mo vs 27.9 mo, *P* = .000016). While it could be that the biology of symptomatic metastases is more aggressive, this study also showed that the median largest lesion size was significantly smaller in the asymptomatic group compared to the symptomatic group (11 mm versus 18 mm, *P* = .0029). In our experience, the larger BMs and their associated perilesional edema can not only contribute to functional decline but often necessitate higher doses of steroids and result therefore in delayed or discontinuous ICI treatment.

Based on our results, we believe that routine screening for BM may improve outcomes for patients. National Comprehensive Cancer Network (NCCN) guidelines recommend considering a brain MRI at mRCC diagnosis as clinically indicated only.^19^ In this study, 28.4% of all mRCC patients had BMs, which is significantly higher than most reports in the literature, most of which range from 8% to 15%^2^^,^^5^^,^^8^ although one other recent study also found that 28.4% of patients developed brain metastases.^3^ In addition, 67.7% of patients developed BM subsequent to first metastatic diagnosis. While these higher rates may reflect improved OS leading to more time for BM development, it also suggests that CNS screening may be increasingly important in longer term survivors and therefore, that screening MRIs therefore should be a standard of care in mRCC, which has been suggested elsewhere. While we screened approximately every 6 months, we cannot make conclusions about the optimal time interval for screening.

The primary limitations of our study are that it is based on a retrospective chart review from a single center experience and, despite its sample size, is still underpowered for some statistical analyses. Thus, we cannot conclude that the observed association between screening for BM and improved OS is causal due to the retrospective nature of our analyses, and further studies are needed to confirm these findings. In addition, being at a large National Cancer Institute-designated cancer center, we routinely receive consultation for more complex cases, especially with advanced intracranial disease. Patients are often treated on clinical trials, which may affect the generalization of our data to the community setting.

Our study is unique in that we present a large cohort of patients with and without BM in the modern treatment era of TKI and ICI for mRCC. We believe that the timeframe from which the data were collected makes it very representative of the current landscape in the management of metastatic ccRCC. We believe the high incidence of BM, especially asymptomatic BM, accurately reflects the modern RCC patient population, which lives much longer. Despite outcomes being worse in patients with BM than without, survival with patients with BM is much better than previously identified and 24% are alive at 5 years. Our data indicate that in addition to the need for patients with BM to undergo aggressive local therapy with SRS and/or craniotomy and avoid delay in systemic therapy, MRI screening should be done at regular intervals.

## Supplementary Material

oyaf387_Supplementary_Data

## Data Availability

The data underlying this article cannot be shared publicly to protect the privacy of individuals whose data are presented in the study. The data will be shared on reasonable request to the corresponding author.
