# Supplementary material for: Survival of patients with metastatic renal cell carcinoma with or without brain metastases
Source: Oncologist. 2025 Nov 25;30(12):oyaf387. doi: 10.1093/oncolo/oyaf387 (PMC12704413; doi:10.1093/oncolo/oyaf387)
Supplement: oyaf387_Supplementary_Data [file oyaf387_supplementary_data.zip › Updated_supplementary_material_Oncologist.docx]

**Supplementary information**

| **TABLE S1.** Comparison of demographics and clinical characteristics between patients treated with ICI (n=247) and those receiving other treatment (n=57) or no systemic treatment (n=34) | | | | | |
| --- | --- | --- | --- | --- | --- |
|  | | **ICI Treatment**  n=247 | **Non-ICI systemic Treatment**  n=57 | **No Systemic Treatment**  n=34 | **p-value** |
| **Age at metastasis diagnosis (years)** | | | |  | **0.01** |
|  | Mean (SD^±^) | 63.9 (11.0) | 63.0 (11.4) | 69.7 (12.5) |  |
| **Gender** | | | |  | 0.1 |
|  | Male | 191 (77.3%) | 42 (73.7%) | 21 (61.8%) |  |
|  | Female | 56 (22.7%) | 15 (26.3%) | 13 (38.2%) |  |
| **Ethnicity** | | | |  | 0.9 |
|  | Non-Hispanic | 225 (91.1%) | 51 (89.5%) | 30 (88.2%) |  |
|  | Hispanic or Latino | 13 (5.3%) | 3 (5.3%) | 3 (8.8%) |  |
|  | Unknown | 9 (3.6%) | 3 (5.3%) | 1 (2.9%) |  |
| **Race** | | | |  | 0.1 |
|  | White or Caucasian | 202 (81.8%) | 48 (84.2%) | 29 (85.3%) |  |
|  | African American | 15 (6.1%) | 4 (7.0%) | 0 (0.0%) |  |
|  | Asian | 6 (2.4%) | 1 (1.8%) | 0 (0.0%) |  |
|  | Unknown | 24 (9.7%) | 4 (7.0%) | 5 (14.7%) |  |
| **Nephrectomy** | | | |  | 0.2 |
|  | Yes | 193 (78.1%) | 41 (71.9%) | 30 (88.2%) |  |
|  | No | 54 (21.9%) | 16 (28.1%) | 4 (11.8%) |  |
| **Grade** | | | |  | **0.03** |
|  | 1 | 5 (2.6%) | 1 (2.7%) | 1 (4.0%) |  |
|  | 2 | 34 (17.9%) | 13 (35.1%) | 11 (44.0%) |  |
|  | 3 | 86 (45.3%) | 11 (29.7%) | 9 (36.0%) |  |
|  | 4 | 65 (34.2%) | 12 (32.4%) | 4 (16.0%) |  |
| **IMDC score^α^** | | | |  | **<0.0001** |
|  | Poor | 46 (18.6%) | 17 (29.8%) | 7 (20.6%) |  |
|  | Intermediate | 149 (60.3%) | 29 (50.9%) | 10 (29.4%) |  |
|  | Favorable | 26 (10.5%) | 1 (1.8%) | 14 (41.2%) |  |
|  | Unknown | 26 (10.5%) | 10 (17.5%) | 3 (8.8%) |  |
| *^±^SD: Standard deviation, ^α^IMDC score: International Metastatic RCC Database Consortium Score* | | | | |  |

| **TABLE S2.** Comparison of demographics and clinical features between patients undergoing screening (n=39) and those diagnosed based on symptom occurrence (n=57) | | | | |
| --- | --- | --- | --- | --- |
|  | | **Screening**  n=39 | **Symptoms**  n=57 | **p-value** |
| **Age at metastasis diagnosis (years)** | | | | 0.4 |
|  | Mean | 60.1 | 61.8 |  |
|  | Median | 59.0 | 61.0 |  |
|  | SD^±^ | 11.0 | 10.2 |  |
| **Gender** | | | | 0.6 |
|  | Male | 31 (79.5%) | 42 (73.7%) |  |
|  | Female | 8 (20.5%) | 15 (26.3%) |  |
| **Ethnicity** | | | | 0.2 |
|  | Non-Hispanic | 39 (100%) | 53 (93.0%) |  |
|  | Hispanic or Latino | 0 | 1 (1.8%) |  |
|  | Unknown | 0 | 3 (5.3%) |  |
| **Race** | | | | **0.03** |
|  | White or Caucasian | 38 (97.4%) | 46 (80.7%) |  |
|  | African American | 1 (2.6%) | 5 (8.8%) |  |
|  | Asian | 0 | 1 (1.8%) |  |
|  | Unknown | 0 | 5 (8.8%) |  |
| **Nephrectomy** | | | | 0.4 |
|  | Yes | 30 (76.9%) | 40 (70.2%) |  |
|  | No | 9 (23.1%) | 17 (29.8%) |  |
| **Grade** | | | | 0.8 |
|  | 1 | 2 (5.1%) | 1 (1.8%) |  |
|  | 2 | 4 (10.3%) | 6 (10.5%) |  |
|  | 3 | 14 (35.9%) | 21 (36.8%) |  |
|  | 4 | 10 (25.6%) | 15 (26.3%) |  |
| **IMDC score^α^** | | | | 0.2 |
|  | Poor | 5 (12.8%) | 16 (28.1%) |  |
|  | Intermediate | 24 (61.5%) | 29 (50.9%) |  |
|  | Favorable | 4 (10.3%) | 3 (5.3%) |  |
|  | Unknown | 6 (15.4%) | 9 (15.8%) |  |
| **Enrolled in a trial prior to BM^Ω^ diagnosis** | | | | 0.5 |
|  | Yes | 5 (12.8%) | 4 (7.0%) |  |
|  | No | 35 (87.2%) | 53 (93%) |  |
| **Recevied ICI*^β^* treatment** | | |  | 0.2 |
|  | Yes | 34 (87.2%) | 43 (75.4%) |  |
|  | No | 5 (12.8%) | 14 (24.6%) |  |
| **Number of BM ^Ω^** | | | | 0.7 |
|  | = 1 | 24 | 32 |  |
|  | > 1 | 15 | 25 |  |
| *^±^SD: Standard deviation, ^α^IMDC score: International Metastatic RCC Database Consortium Score. ^β^ICI: immune checkpoint inhibitors. ^Ω^BM***:** *brain* *metastases* | | | | |

| **TABLE S3.** Logistic Multivariate Regression Analyses of Brain Metastases | | | |  |
| --- | --- | --- | --- | --- |
|  | | **Odds Ratio (95% CI)** | **p-value** |  |
| **Age at metastasis diagnosis** | | 0.9 (0.9-1) | **0.005** |  |
| **Gender** | | |  |  |
|  | Male (n=254) | Reference | |  |
|  | | Female (n=84) | 1.1 (0.5 – 2.4) | 0.7 |
| **Ethnicity** | |  |  |  |
|  | Non-Hispanic (n=306) | Reference | |  |
|  | Hispanic (n=19) | 0.2 (0.03 – 2.5) | 0.2 |  |
|  | Unknown (n=13) | NA | 1.0 |  |
| **Race** | |  |  |  |
|  | White or Caucasian (n=279) | Reference | |  |
|  | Black or African American (n=19) | 0.0 (0.0 – NA) | 0.9 |  |
|  | Other (n=40) | 0.8 (0.3 – 2.4) | 0.8 |  |
| **Nephrectomy** | | |  |  |
|  | Yes (n=264) | Reference | |  |
|  | | N0 (n=74) | 0.7 (0.1 – 3.3) | 0.6 |
| **Grade** | |  |  |  |
|  | Grade 1 (n=7) | Reference | |  |
|  | Grade 2 (n=58) | 0.5 (0.05 – 6.7) | 0.6 |  |
|  | Grade 3 (n=106) | 0.9 (0.09 – 10.3) | 0.9 |  |
|  | Grade 4 (n=81) | 0.8 (0.08 - 9.5) | 0.9 |  |
| **ICIs^β^ treatment** | | |  |  |
|  | Yes (n=247) | Reference | |  |
|  | | No (n=91) | 1.3 (0.6 – 3.1) | 0.4 |
| **IMDC Score*** | |  |  |  |
|  | Poor (n=70) | Reference | |  |
|  | Intermediate (n=188) | 0.8 (0.3-1.9) | 0.6 |  |
|  | Favorable (n=41) | 0.5 (0.1-1.8) | 0.3 |  |
| *^β^ICI^:^ Immune check-point inhibitors, *IMDC score: International Metastatic RCC Database Consortium Score*  *NA: Not applicable; invalid results due to extreme outliers* | | | |  |

**Supplementary Figures**

Figure S1. Flow chart of patient selection.

Figure S2. Overall survival of all patients. The gray area represents the 95% confidence interval.

Figure S3. Overall survival of patients by IMDC score.

Figure S4. Time from mRCC to development of brain metastasis in patients who had screening brain MRI versus those who did not, among those who developed brain metastases after the development of other metastatic disease.

Figure S5. Kaplan–Meier overall survival of patients with a single BM at diagnosis compared with those with more than one BM at diagnosis.

Figure S6. Kaplan–Meier overall survival of patients treated with SRS only, WBRT only or both.

Figure S7. Kaplan–Meier overall survival of treated with SRS vs patients treated with WBRT.


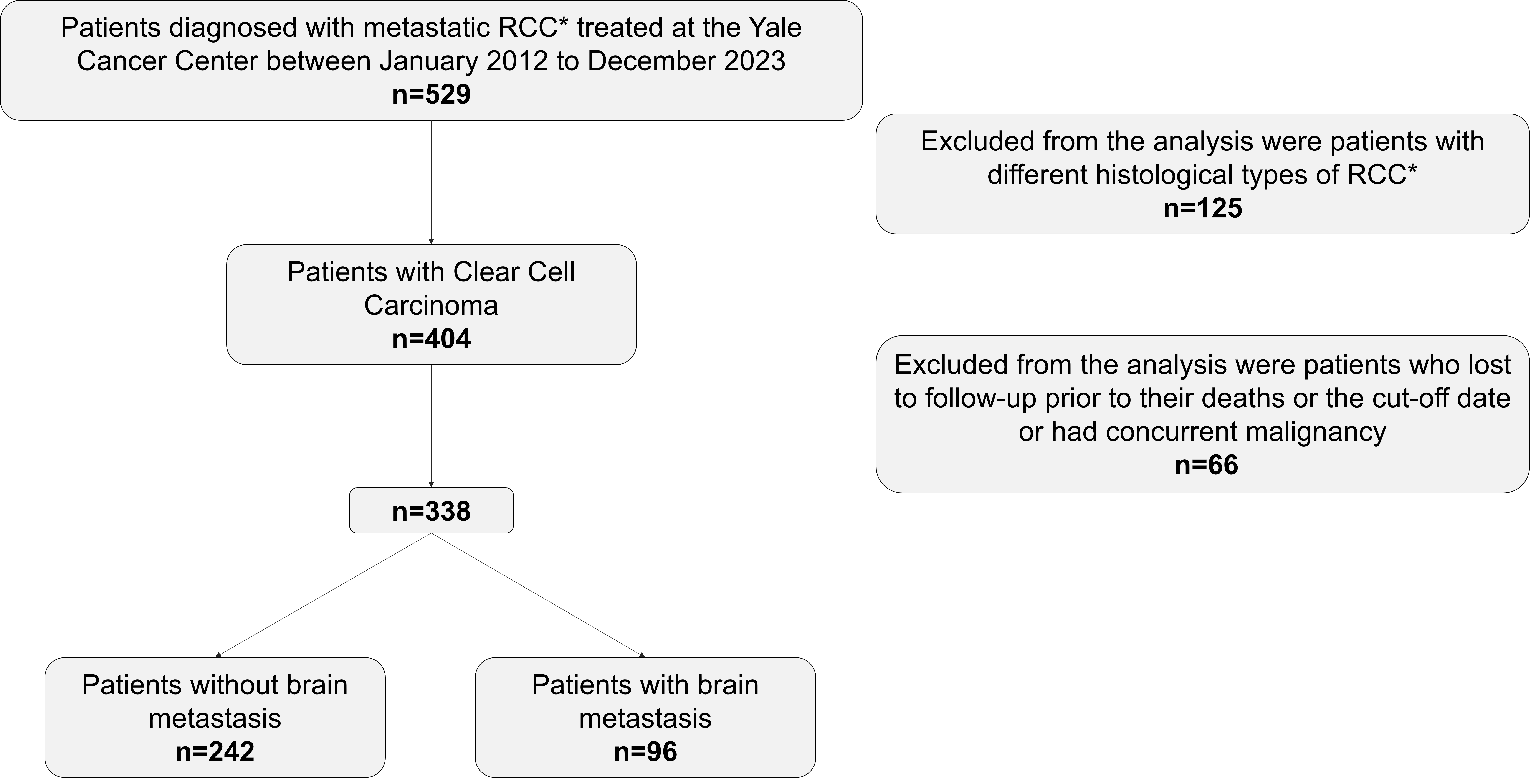


Figure S1. Flow chart of patient selection.


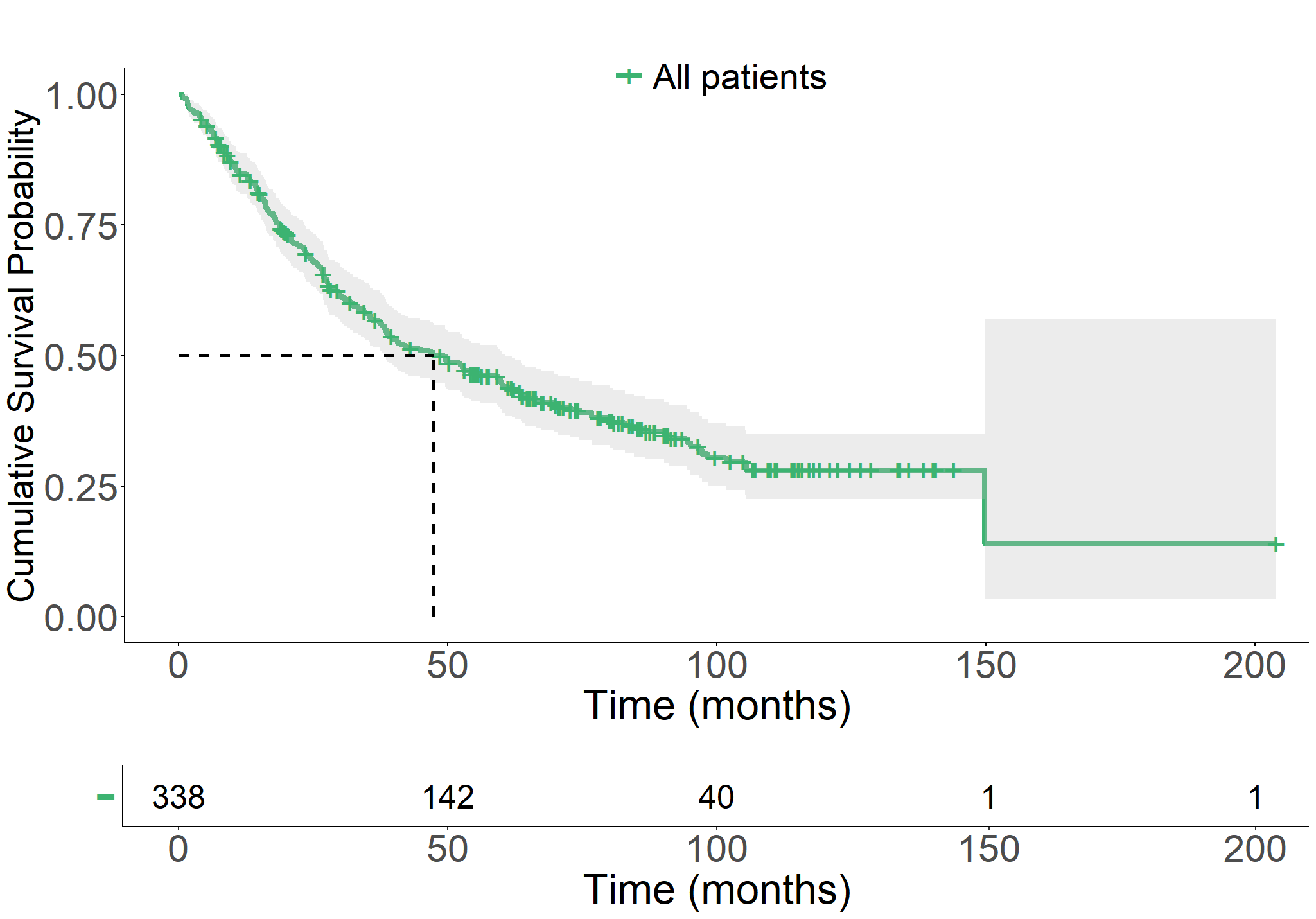


Figure S2. Overall survival of all patients. The gray area represents the 95% confidence interval.


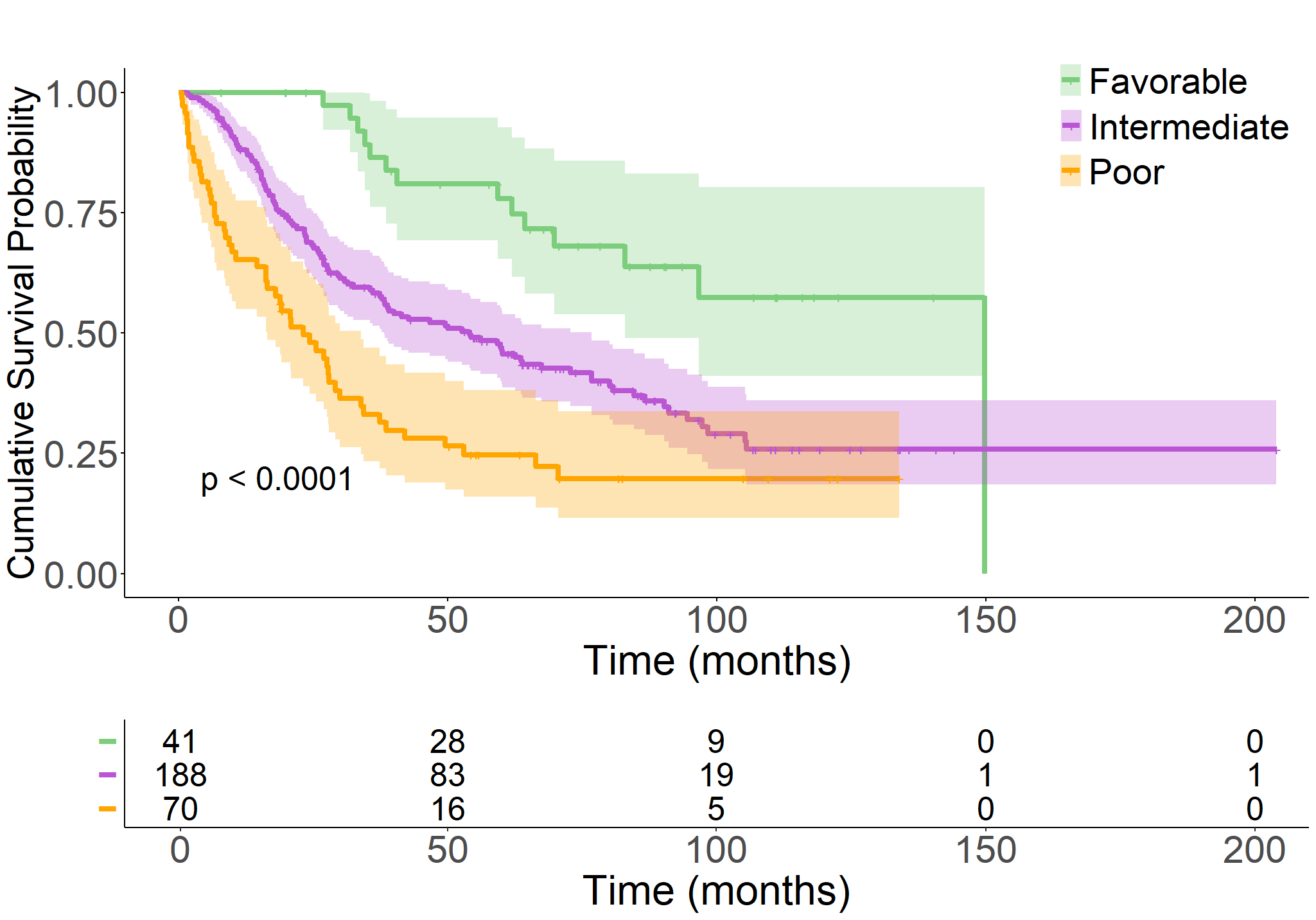


Figure S3. Overall survival of patients by IMDC score


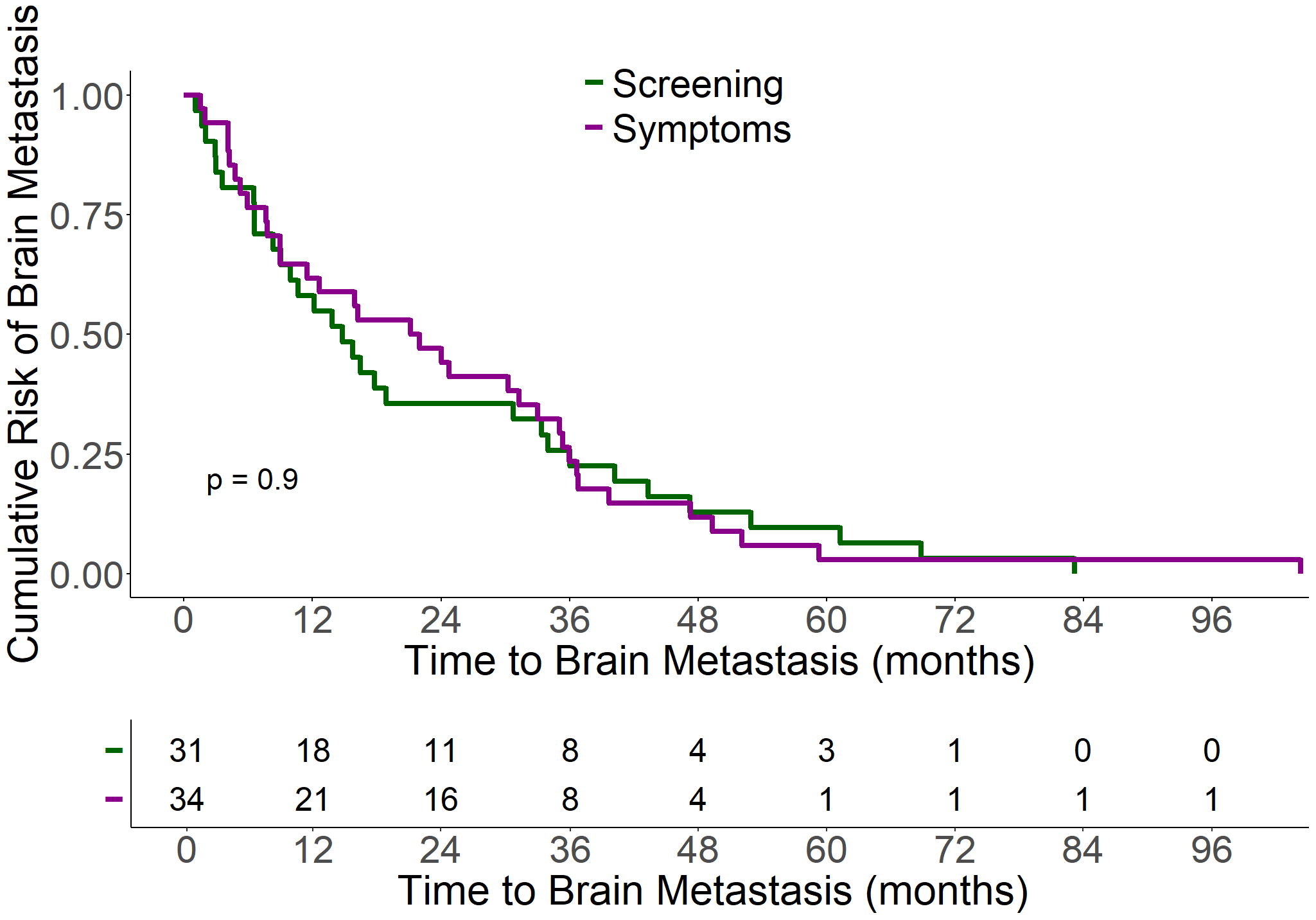


Figure S4. Time from mRCC to development of brain metastasis in patients who had screening brain MRI versus those who did not, among those who developed brain metastases after the development of other metastatic disease.


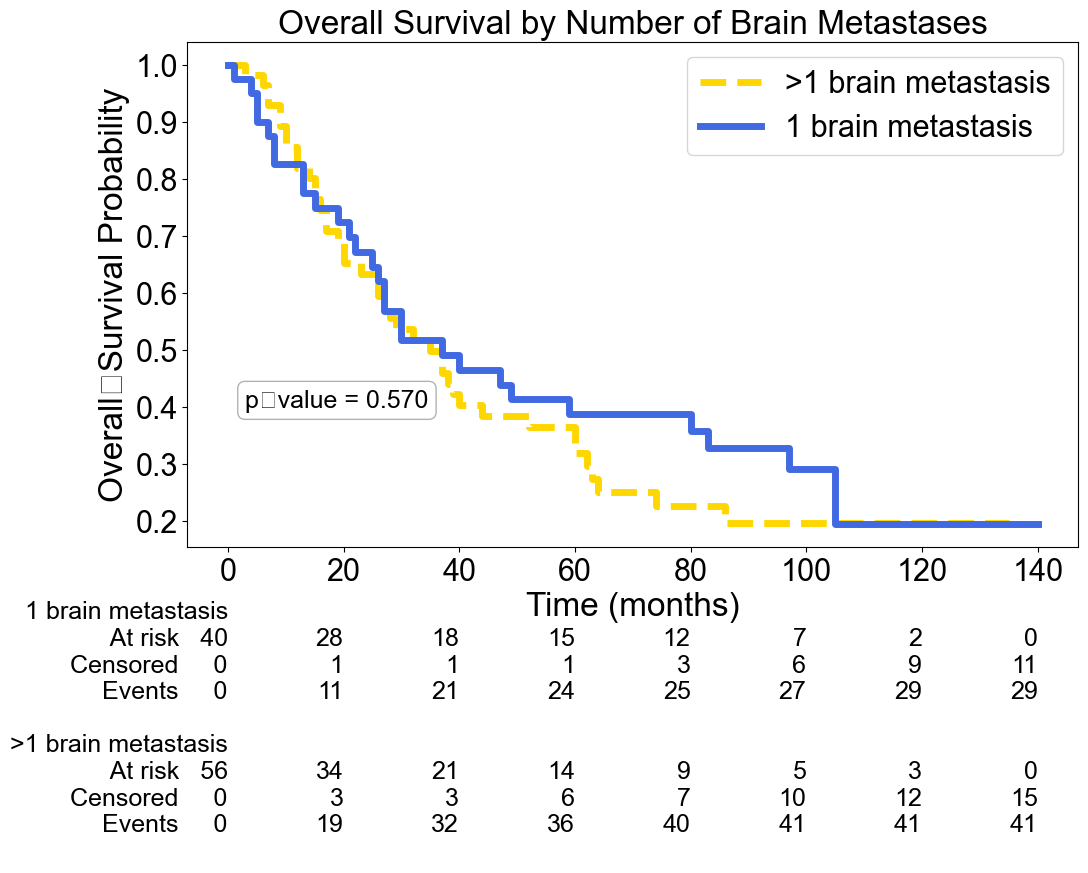

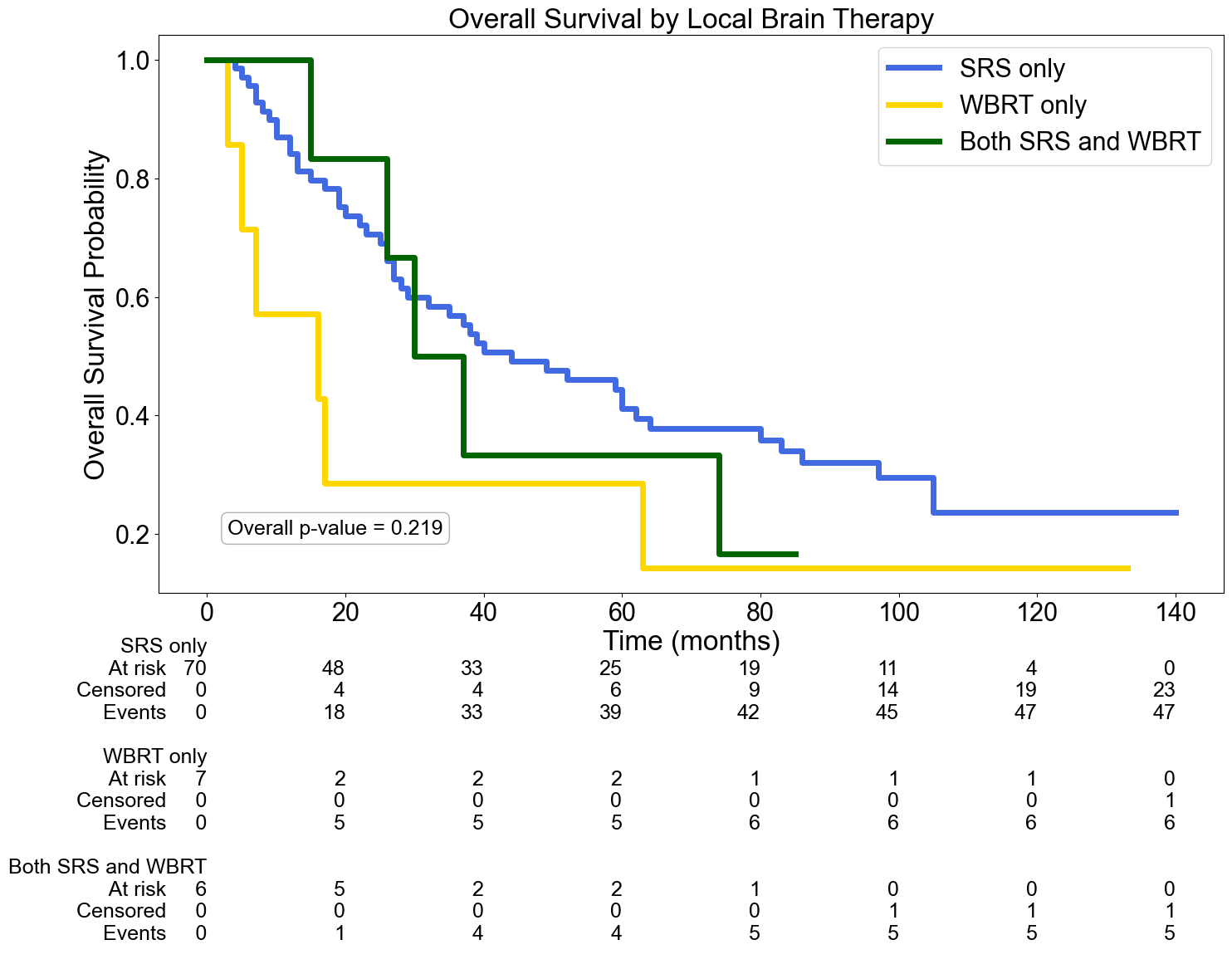

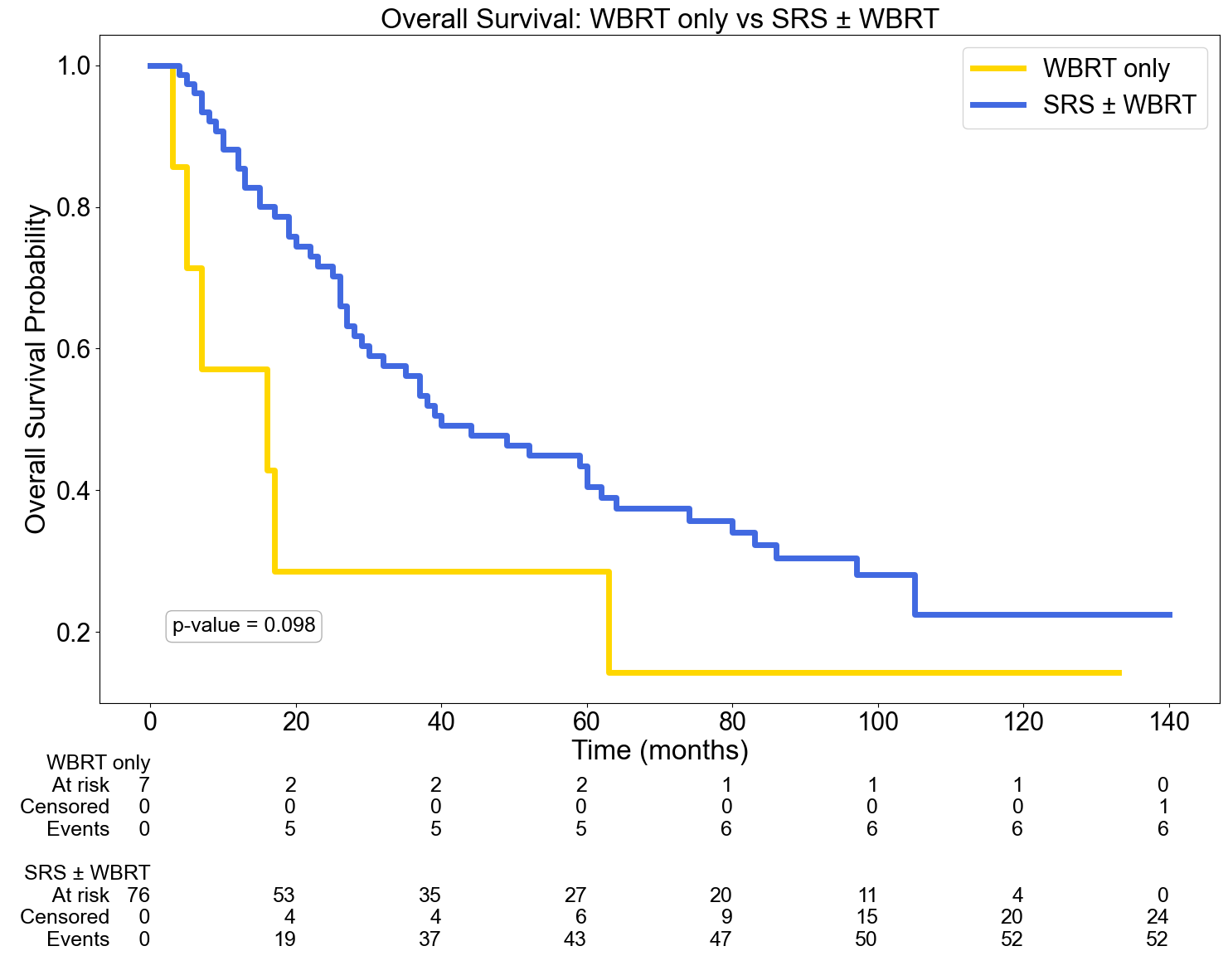


Figure S5. Kaplan–Meier overall survival of patients with a single BM at diagnosis compared with those with more than one BM at diagnosis.

Figure S6. Kaplan–Meier overall survival of patients treated with SRS only, WBRT only or both.

Figure S7. Kaplan–Meier overall survival of treated with SRS vs patients treated with WBRT.
