# Supplementary figures and images for: Survival of patients with metastatic renal cell carcinoma with or without brain metastases
Source: Oncologist. 2025 Nov 25;30(12):oyaf387. doi: 10.1093/oncolo/oyaf387 (PMC12704413; doi:10.1093/oncolo/oyaf387)

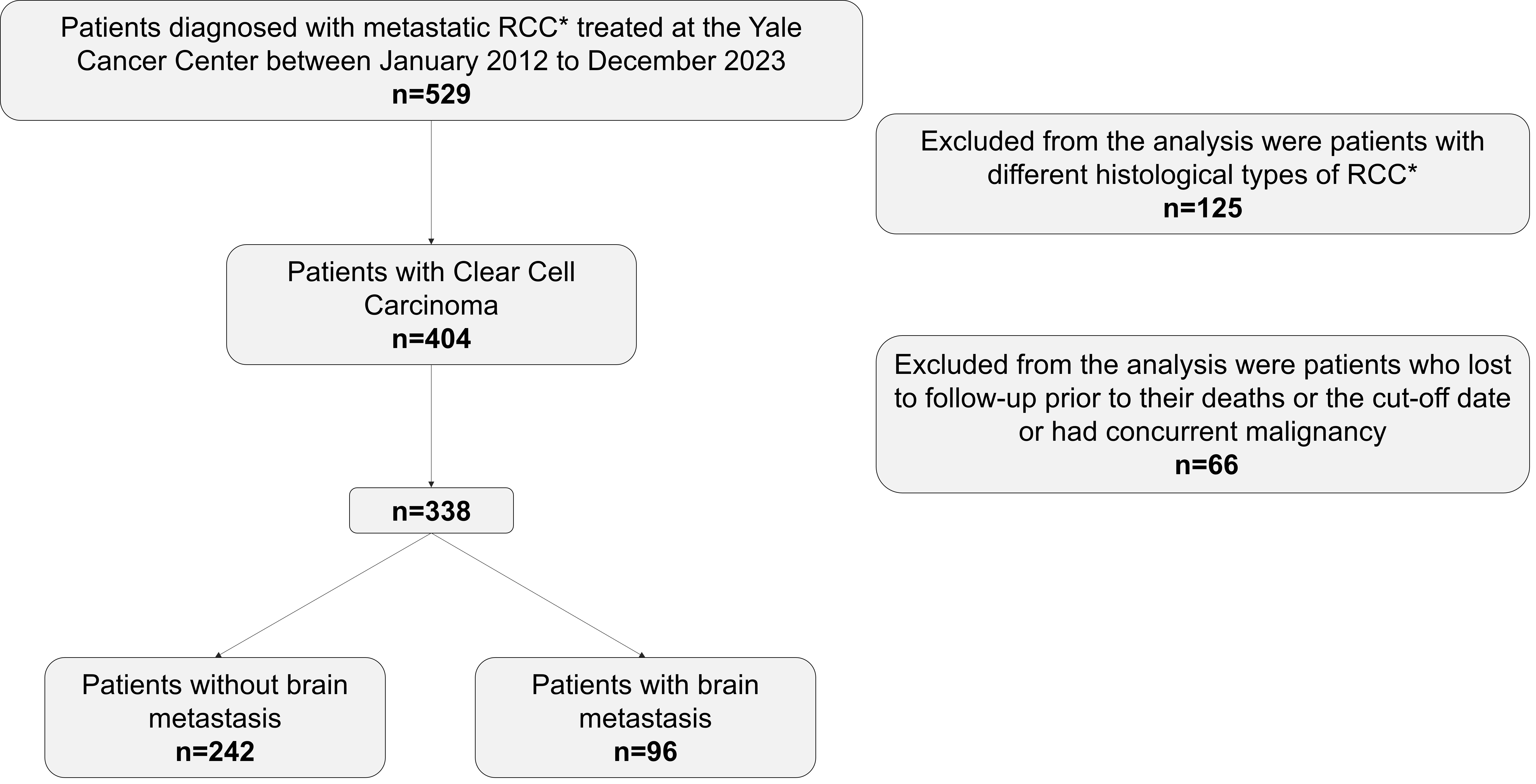

Supplement: oyaf387_Supplementary_Data [file oyaf387_supplementary_data.zip › Figure S1.png]

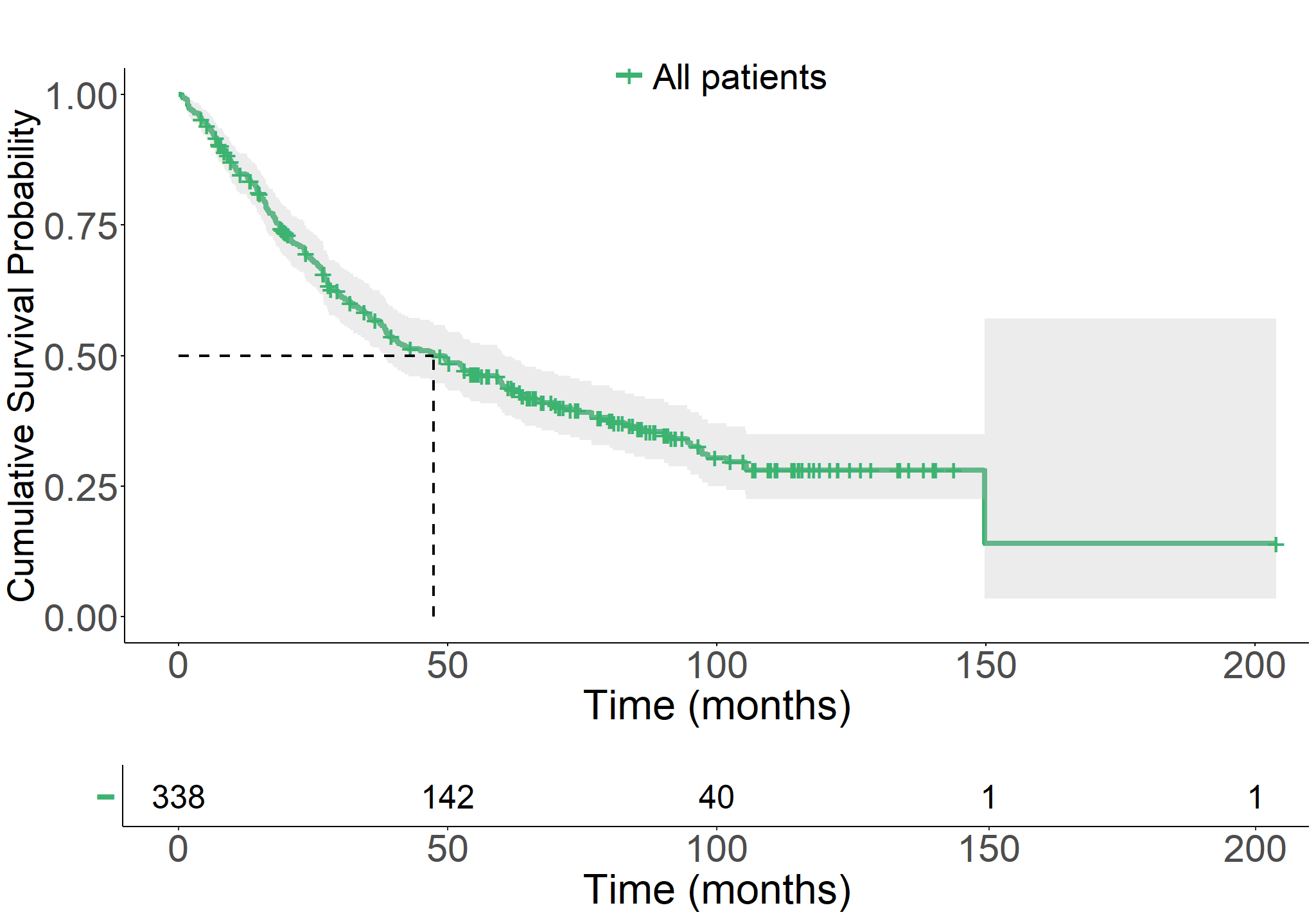

Supplement: oyaf387_Supplementary_Data [file oyaf387_supplementary_data.zip › Figure S2.tiff]

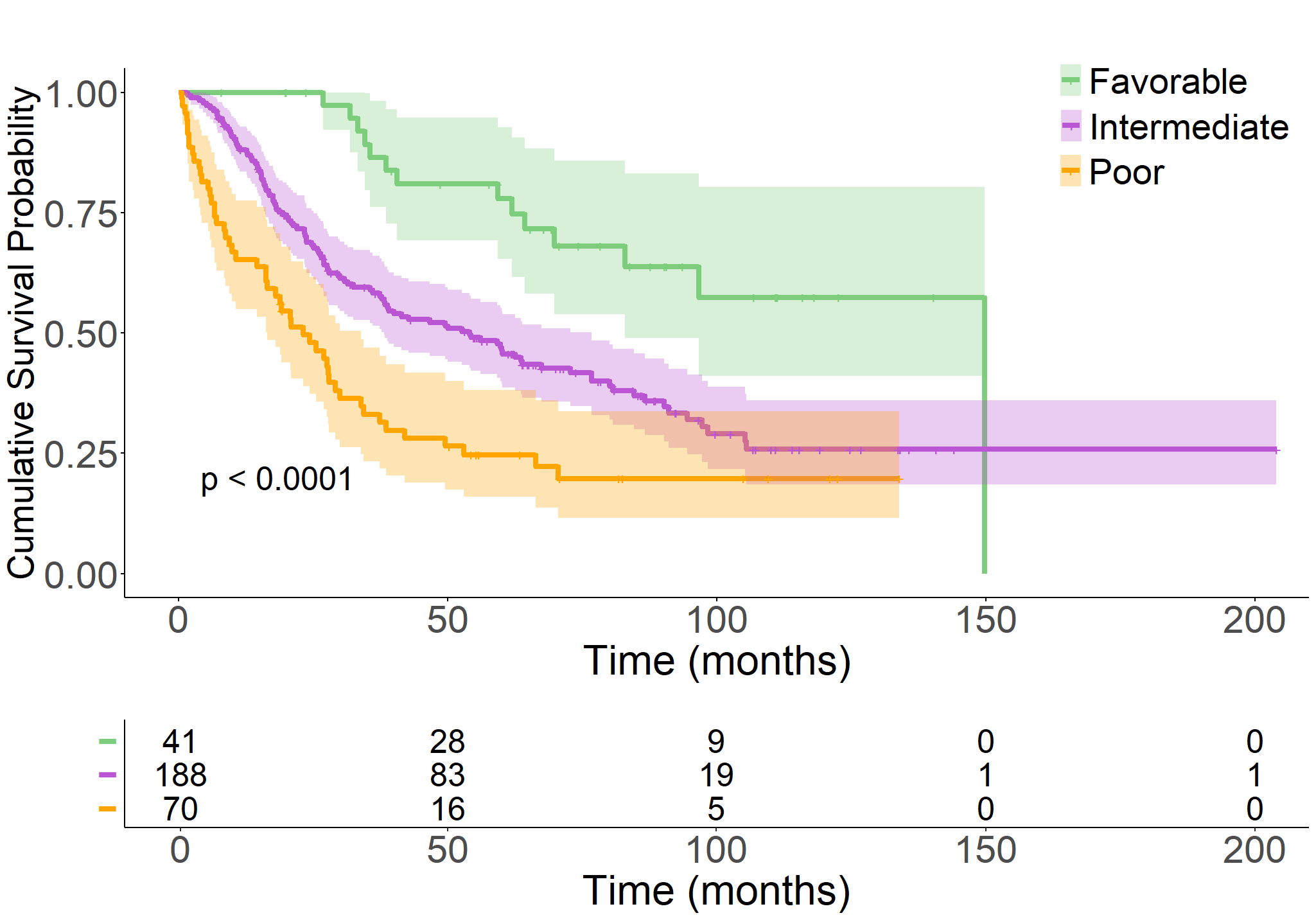

Supplement: oyaf387_Supplementary_Data [file oyaf387_supplementary_data.zip › Figure S3.tiff]

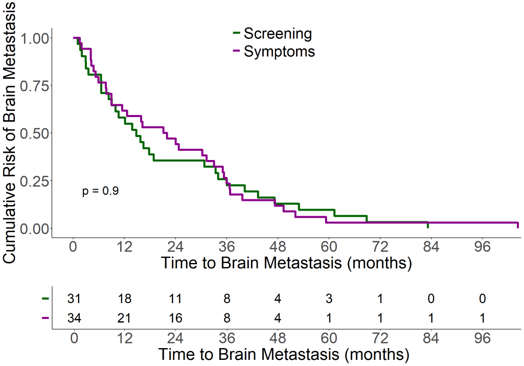

Supplement: oyaf387_Supplementary_Data [file oyaf387_supplementary_data.zip › Figure S4.tif]

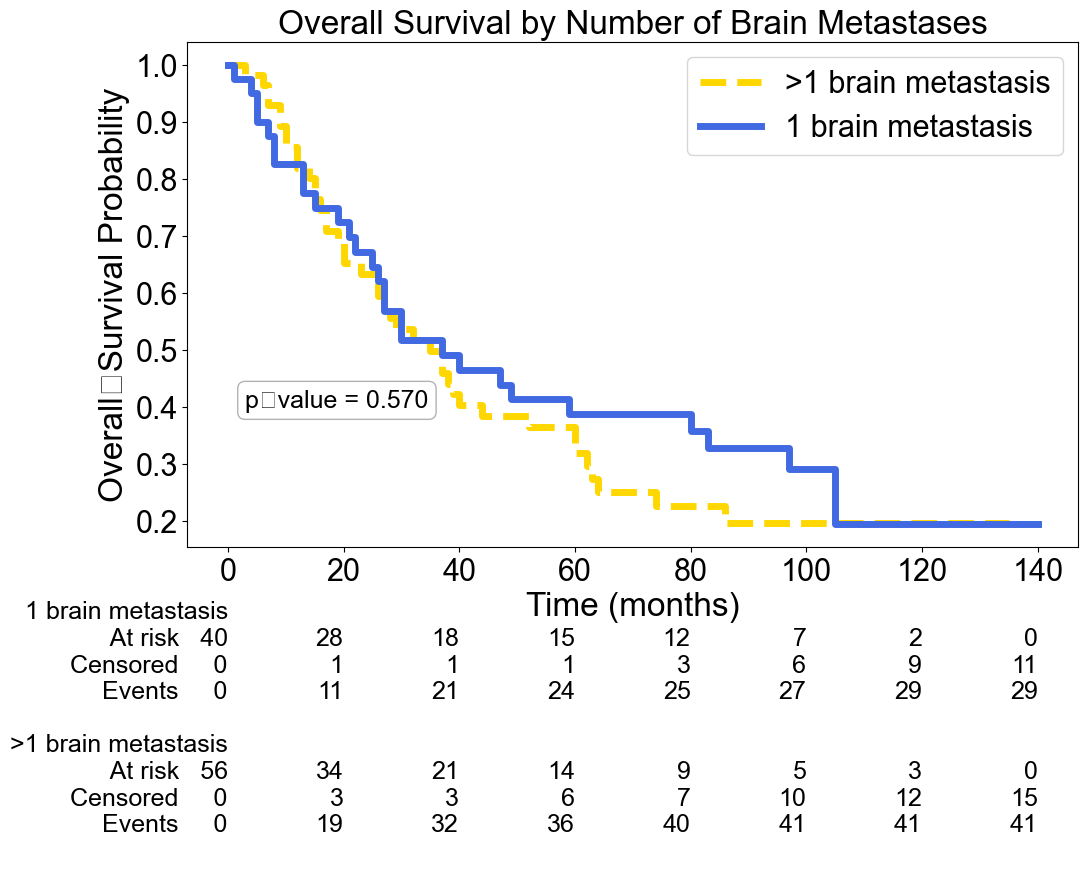

Supplement: oyaf387_Supplementary_Data [file oyaf387_supplementary_data.zip › Figure S5.png]

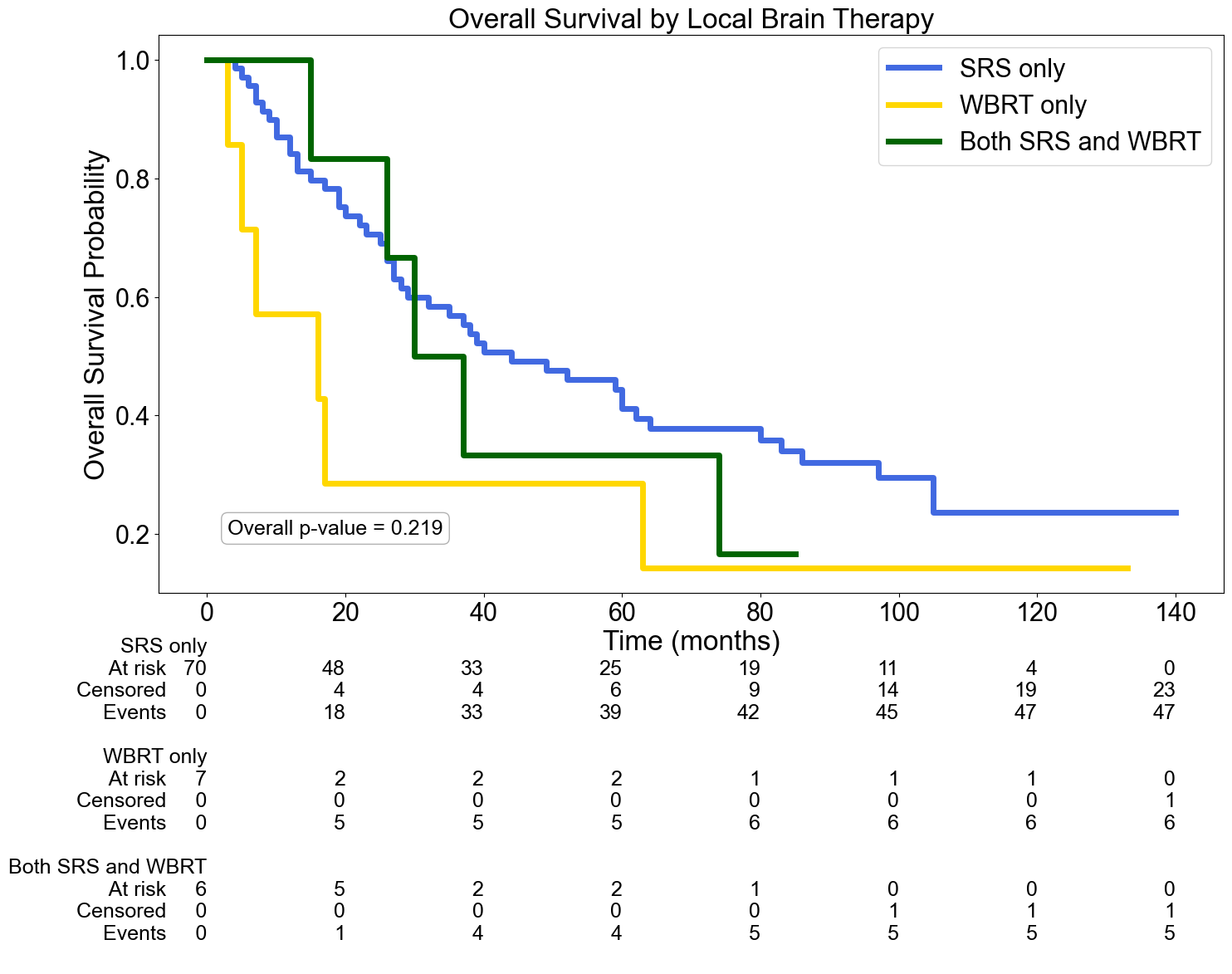

Supplement: oyaf387_Supplementary_Data [file oyaf387_supplementary_data.zip › Figure S6.png]

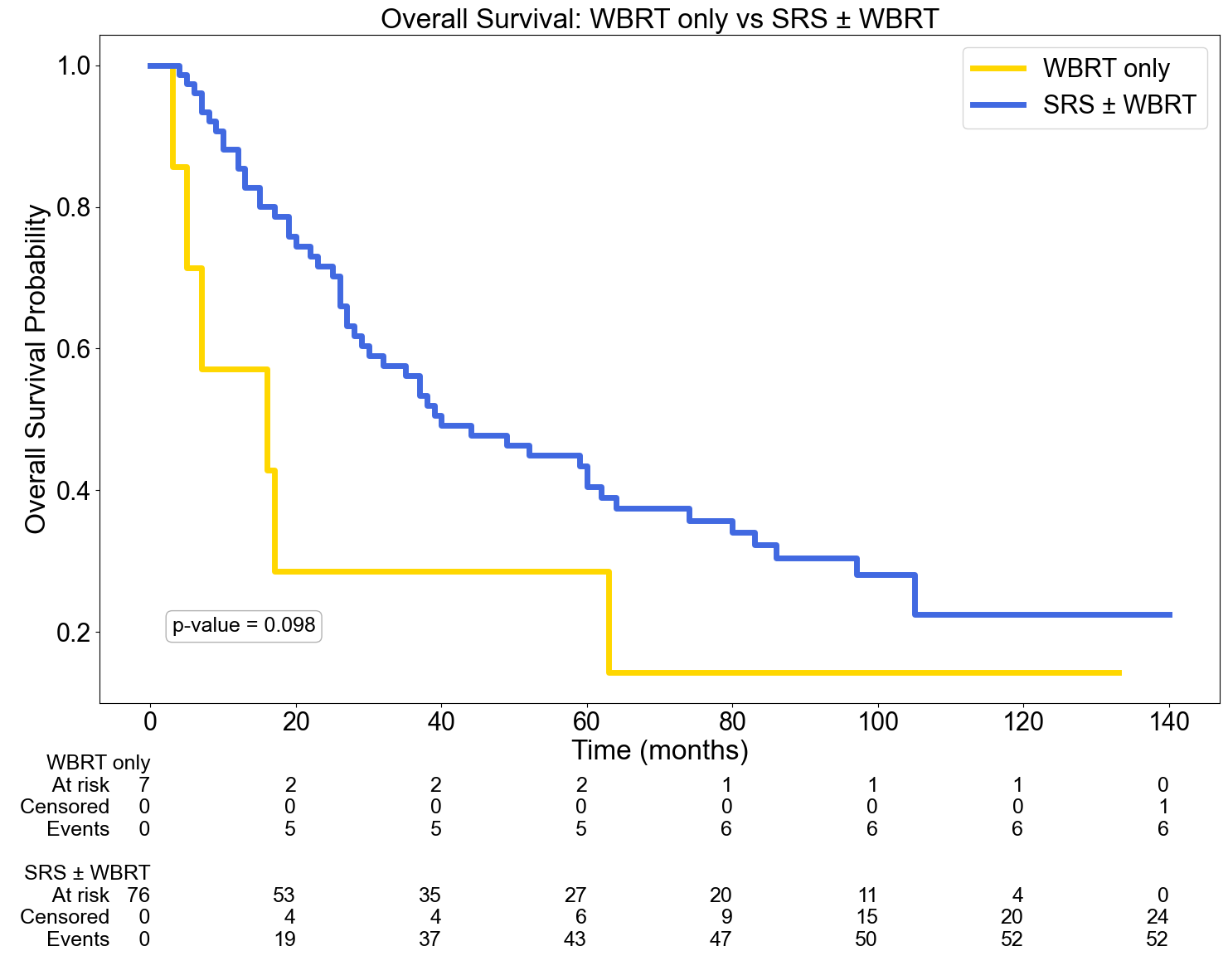

Supplement: oyaf387_Supplementary_Data [file oyaf387_supplementary_data.zip › Figure S7.png]
